# Association of Different Malnutrition Parameters and Clinical Outcomes among COVID-19 Patients: An Observational Study

**DOI:** 10.3390/nu14163449

**Published:** 2022-08-22

**Authors:** Claudia Gregoriano, Manyola Voelkle, Daniel Koch, Stephanie Isabelle Hauser, Alexander Kutz, Beat Mueller, Philipp Schuetz

**Affiliations:** 1 Medical University Department of Medicine, Kantonsspital Aarau, 5001 Aarau, Switzerland; 2 Department of Clinical Research, University Hospital Basel, University of Basel, 4056 Basel, Switzerland

**Keywords:** nutritional risk screening 2002, body mass index, albumin, COVID-19, in-hospital outcomes

## Abstract

**Background**: Malnutrition is highly prevalent in medical inpatients and may also negatively influence clinical outcomes of patients hospitalized with COVID-19. We analyzed the prognostic implication of different malnutrition parameters with respect to adverse clinical outcomes in patients hospitalized with COVID-19. **Methods**: In this observational study, consecutively hospitalized adult patients with confirmed COVID-19 at the Cantonal Hospital Aarau (Switzerland) were included between February and December 2020. The association between Nutritional Risk Screening 2002 (NRS 2002) on admission, body mass index, and admission albumin levels with in-hospital mortality and secondary endpoints was studied by using multivariable regression analyses. **Results**: Our analysis included 305 patients (median age of 66 years, 66.6% male) with a median NRS 2002-score of 2.0 (IQR 1.0, 3.0) points. Overall, 44 patients (14.4%) died during hospitalization. A step-wise increase in mortality risk with a higher nutritional risk was observed. When compared to patients with no risk for malnutrition (NRS 2002 < 3 points), patients with a moderate (NRS 2002 3–4 points) or high risk for malnutrition (NRS 2002 ≥ 5 points) had a two-fold and five-fold increase in risk, respectively (10.5% vs. 22.7% vs. 50.0%, *p* < 0.001). The increased risk for mortality was also confirmed in a regression analysis adjusted for gender, age, and comorbidities (odds ratio for high risk for malnutrition 4.68, 95% CI 1.18 to 18.64, *p* = 0.029 compared to patients with no risk for malnutrition). **Conclusions**: In patients with COVID-19, the risk for malnutrition was a risk factor for in-hospital mortality. Future studies should investigate the role of nutritional treatment in this patient population.

## 1. Introduction

Malnutrition is highly prevalent in hospitalized patients. In particular, in internal medicine wards, rates of up to 30–50% are reported, indicating that this issue represents an important public health problem [1,2,3,4]. Malnutrition is known to cause a longer length of hospital stay (LOS) [4] and higher mortality in hospitalized patients [5,6]. Across patients hospitalized with coronavirus disease 2019 (COVID-19), gastrointestinal symptoms such as nausea, vomiting, or loss of appetite are common [7,8]. Moreover, other symptoms that are associated with COVID-19, such as dysgeusia and respiratory problems, may lead to a decreased food intake and altogether deteriorating nutritional status [9]. Thus, patients with COVID-19 may have an increased risk for poor food intake and malnutrition. This is also shown in data from France, reporting that up to 40% of hospitalized patients with COVID-19 are at risk for malnutrition [10], with a significantly higher prevalence among patients transferred from the intensive care unit (ICU) to a normal ward [11].

In order to access the nutritional risk, different screening tools are available [6,12]. Among these, the Nutritional Risk Screening 2002 (NRS 2002) is an established and validated tool, and is recommended by the European Society for Clinical Nutrition and Metabolism (ESPEN) for the screening of nutritional risk [12,13,14]. Further, it has been shown to be a good predictor for adverse outcomes among inpatients [5,15]. A recent meta-analysis showed that NRS 2002 can also be used as a good predictor for nutritional risk among inpatients with COVID-19 [16] and correlates with a longer LOS [17,18,19] and mortality [18,20]. In addition to nutritional risk scores, other nutritional markers have also been associated with adverse clinical outcomes among medical [21,22] and COVID-19 patients, such as obesity [23] and low albumin levels, which are both independently correlated with ICU admission [11] and a more severe course of COVID-19 [24]. Still, there is a lack of studies comparing the different nutritional parameters regarding their association with adverse clinical outcomes among COVID-19 patients in order to better risk stratify patients. The aim of this study was to assess the association between the nutritional risk status on admission, based on the NRS 2002-score, body mass index (BMI), and serum albumin levels during hospitalization, with in-hospital mortality, ICU admission, and LOS.

## 2. Materials and Methods

### 2.1. Study Design and Setting

This observational study included consecutively hospitalized adult patients (≥18 years) with confirmed severe acute respiratory syndrome coronavirus type 2 (SARS-CoV-2) infection and with a LOS of at least 24 h at the Cantonal Hospital Aarau (Switzerland) between 26 February 2020 and 31 December 2020. This study was reviewed and approved by the local ethics committee (EKZN, 2020-01306).

Detailed description of the study methodology has been previously reported [25]. During the first wave, a confirmed SARS-CoV-2 infection was defined as combination of typical clinical symptoms, such as respiratory symptoms with or without fever, and/or pulmonary infiltrates and/or anosmia/dysgeusia and a positive real-time reverse transcription polymerase chain reaction (RT-PCR) test, obtained from nasopharyngeal swabs or lower respiratory tract samples, according to World Health Organization (WHO) guidance [26,27]. During the second wave, more asymptomatic or oligosymptomatic patients with positive RT-PCR tests were hospitalized due to non-COVID-19 reasons, such as childbirth or trauma. Rapid antigen testing was authorized by the Federal Office of Public Health in Switzerland in November 2020. Therefore, data for the second wave also include patients with positive rapid antigen tests compared to the first wave. Due to the lower predictive value for asymptomatic cases, we excluded patients without symptoms unless their rapid antigen results were confirmed by a positive RT-PCR test. Further, patients refusing the general informed consent and patients without an available NRS 2002-score at admission were excluded from this analysis.

### 2.2. Data Collection

All analyzed data were collected as part of the clinical routine during the hospital stay (from admission to discharge or death). Chart reviews and automatic export from electronic health records (EHR) were performed in order to identify vital signs and clinical characteristics upon admission, as well as sociodemographic factors and comorbidities based on pre-existing diagnoses. The age-adjusted Charlson comorbidity index (ACCI) [28] and the Clinical Frailty Scale score (CFS) [29] were calculated for all patients as part of the clinical routine or through chart review. Nutritional risk screening was performed using the NRS 2002 within the first 24 h of admission. Laboratory values were available according to clinical routine and corresponded to first blood draw obtained within 24 h from admission.

### 2.3. Endpoint and Study Objective

The primary endpoint of this study was all-cause in-hospital mortality. Secondary endpoints were ICU admission and LOS. All defined endpoints were verified through chart review.

### 2.4. Statistical Analysis

Discrete variables are expressed as frequency (percentage) and continuous variables as medians with interquartile ranges (IQR) or mean with standard deviation (SD). Wilcoxon rank-sum test was applied to compare continuous variables and the Pearson’s chi-squared test was applied to compare categorical or binary variables. Association of NRS 2002, BMI and albumin with the primary and secondary endpoints was analyzed by performing logistic regression for binary dependent variables and ordinary least-squares linear regression for continuous variable. For the calculation of the LOS, we excluded patients who died during hospitalization. Cut-offs were set as follows: NRS 2002 at nutritional risk was defined as ≥3 points according to Kondrup [30]. Further, 4 points indicated moderate risk and ≥5 points indicated high risk for malnutrition. Cut-off for BMI was <20 kg/m^2^ if age was <70 years and <22 kg / m^2^ if age was ≥70 years [31]. Cut-off values for albumin levels were set at <34 g/L, according to the reference range at the Cantonal Hospital Aarau. Odds ratios (OR) and regression coefficients were calculated with corresponding 95% confidence intervals (CI) and *p*-values as measures of association. As predefined and in order to not over-adjust the model, regression models were adjusted for gender and ACCI (adjusted model). Thus, gender, age, and a relevant number of comorbidities were considered for the adjustment. Moreover, we also investigated subgroups for differences in performance based on socio-demographic factors (age and sex), comorbidities, ACCI, and CFS for the primary endpoint. For the subgroup analysis, a NRS 2002 < 3 points was considered low and NRS 2002 ≥ 3 points high risk for malnutrition. A two-sided *p*-value of <0.05 was considered significant. Statistical analysis was performed using Stata 15.1 (StataCorp, College Station, TX, USA).

## 3. Results

### 3.1. Characteristics of the Study Population

**Figure 1:**
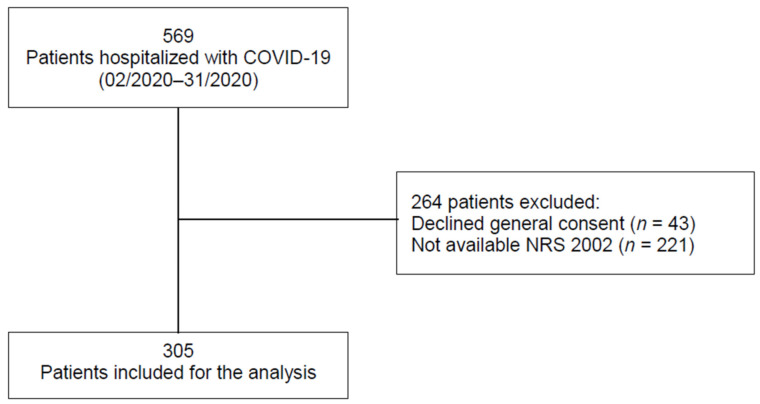
Study flowchart. A total of 305 patients were included in the final analysis. NRS 2002, nutritional risk screening 2002.

Overall, 305 COVID-19 patients were included in the study. Figure 1 provides an overview of the study flow. Baseline characteristics in the overall cohort and stratified data according to the primary endpoint are summarized in Table 1. The median age was 66 years (IQR 55.0, 75.0), and 66.6% were male. Patients had a high burden of comorbidities with a median ACCI of 3 points (IQR 2.0, 5.0) and a median CFS of 3 points (IQR 2.0, 4.0). The most prevalent comorbidities included hypertension (54.8%, *n* = 167), obesity (32.1%, *n* = 97), diabetes (26.6%, *n* = 81), chronic kidney disease (CKD) (22.3%, *n* = 68), and coronary artery disease (20.7%, *n* = 63). The median BMI was 27.6 kg/m^2^ (IQR 24.2, 31.7), indicating overweight in the overall study population. Overall, 14.4% (*n* = 44) were admitted to ICU, with 7.2% (*n* = 22) needing mechanic ventilation support. Median LOS was 7.0 days (IQR 4.0, 13.0) and 14.4% (*n* = 44) died during hospitalization. In total, 76 patients (25%) were at risk for malnutrition (NRS 2002 ≥ 3 points). Compared to patients with low risk for malnutrition (NRS 2002 < 3 points), patients with a moderate (NRS 2002 3–4) or high risk for malnutrition (NRS 2002 ≥ 5) had a significant higher risk for in-hospital mortality (10.5% vs. 22.7% vs. 50.0%, *p* < 0.001).

### 3.2. Association of NRS 2002-, BMI-, and Albumin Categories and the Primary Endpoint

Higher NRS 2002-scores were associated with higher odds for in-hospital mortality. An increase of 1 point in the NRS 2002-score was associated with a 39% higher risk for in-hospital mortality with an adjusted OR of 1.39 (95% CI 1.07, 1.80, *p* = 0.013) (Table 2). Patients with an NRS 2002-score ≥ 5 points had a 4.68-fold higher risk (95% CI 1.18, 18.64, *p* = 0.029) for in-hospital mortality compared to patients with an NRS 2002-score < 3 points. However, this was not significant for patients with an NRS score of 3–4 points. Median albumin levels were significantly lower in non-survivors vs. survivors (28.0 g/L vs. 30.5 g/L, *p* < 0.01) and higher albumin levels were associated with a lower risk for the primary endpoint (adjusted OR 0.92 (95% CI 0.85, 0.99, *p* = 0.030)). Among our cohort, the median BMI at admission was in the overweight range, with 27.6 kg/m^2^, and 97 patients had obesity as a comorbidity. For BMI, no significant association with the primary outcome was found.

### 3.3. Association of NRS 2002-, BMI- and Albumin Categories and Secondary Endpoints

**Figure 2:**
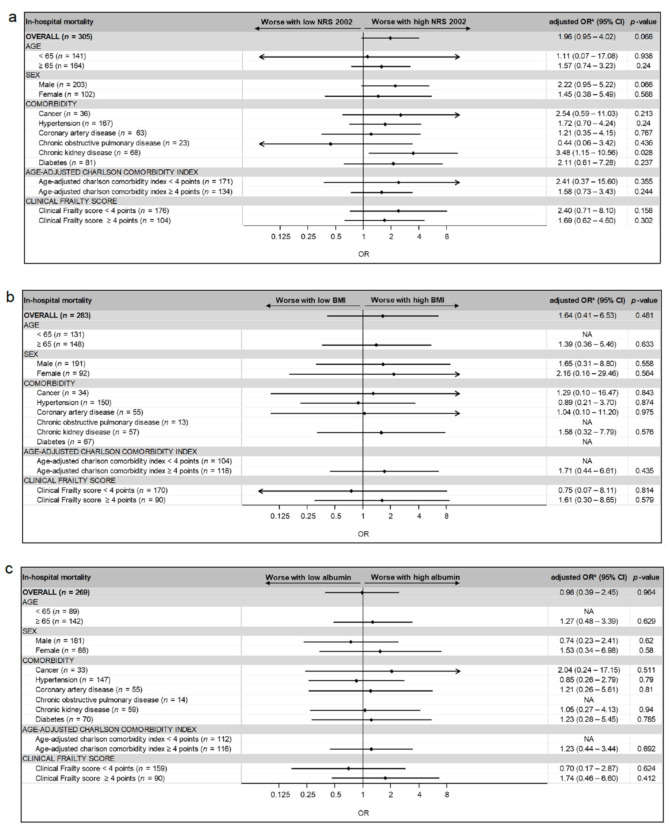
Adjusted odds ratios in-hospital mortality stratified by (**a**) NRS 2002 < 3 vs. NRS 2002 ≥ 3; (**b**) BMI < 20 if age < 70, BMI < 22 if age ≥ 70 vs. BMI ≥ 20 if age < 70, BMI ≥ 22 if age ≥ 70; (**c**) albumin levels < 34.0 g/L vs. ≥34.0 g/L in pre-specified subgroups. BMI, body mass index; NRS 2002, nutritional risk screening 2002; OR, odds ratio; CI, confidence interval, NA, not applicable. * adjusted for gender and age-adjusted Charlson comorbidity index (ACCI).

Higher levels of albumin were associated with a lower risk for ICU admission and shorter LOS (adjusted OR 0.88 (IQR 0.82, 0.95), *p* = 0.001, adjusted coefficient −0.34 days per 1 g/l increase in albumin concentrations (IQR −0.52, −0.17, *p* < 0.001, respectively)) (Table 3 and Table 4). In the subgroup analysis, for the group of patients with CKD, a NRS 2002-score ≥ 3 points was associated with higher odds for in-hospital mortality (adjusted OR 3.48 (IQR 1.15, 10.56), *p* = 0.028), whereas this was not shown in the other subgroups (Figure 2). No significant association was found for the BMI with the secondary endpoints.

## 4. Discussion

In this observational study, we assessed the association of nutritional risk with in-hospital mortality, ICU admission, and LOS in patients hospitalized with COVID-19. We found that a higher nutritional risk as assessed through NRS 2002 in patients with COVID-19 was associated with a higher risk for in-hospital mortality, with a five-fold increase in mortality in highest risk patients compared to patients with no nutritional risk. However, this association was not significant for patients with a moderate NRS score of 3 to 4 points. Similar associations were not found for the risk of ICU admission or longer LOS. While lower albumin levels were associated with a higher risk for in-hospital mortality, ICU admission, and longer LOS, no associations were found for different levels of BMI.

When compared to other studies assessing the nutritional risk with NRS 2002, the prevalence of patients at risk for malnutrition was only 25% in our cohort, and thus lower compared to other reports, where prevalences of up to 90% were reported [17,19]. These differences may be partly explained by selection bias in the different cohorts. In one study from China, patients were transferred from other hospitals to the study hospital; thus, the overall severity of COVID-19 was very high and, additionally, only patients over 65 years were included, both contributing to more NRS 2002 points [17]. In our analysis, the nutritional risk expressed by the NRS 2002 was associated with a higher risk of in-hospital mortality, which was already shown in a study with patients hospitalized in a medical ward and ICU with COVID-19 [32] and in a cohort of severely and critically ill patients with COVID-19 [18]. However, these associations are not only COVID-19-specific, but generally true for medical inpatients with and without infections [33,34].

In contrast to other studies, we did not find an association of NRS 2002 with ICU admission [35] or LOS [19,36]. Importantly, due to restricting intensive care to younger and healthier patients during the pandemic, some patients with a theoretical need for ICU admission may not have been admitted, which introduces bias to the analysis. Also for LOS, some old and frail patients may have been transferred to lower-acuity hospitals, again introducing bias to the analysis.

In the subgroup analysis, we found that, among patients with a CKD, an NRS 2002-score ≥ 3 points was associated with a higher risk for in-hospital mortality. In patients with CKD, protein-energy wasting is common [37] and is associated with disease progression and mortality [38]. A recent study described an association of an NRS 2002-score ≥ 3 with in-hospital mortality also in hospitalized patients with CKD [39]. Further, in our study, creatinine levels of surviving and deceased patients differed significantly, indicating worse renal function in the deceased patients. Moreover, the prevalence of CKD was higher in non-survivors vs. survivors. Similar to these findings, an analysis from over 17 million adults in the United Kingdom (UK) reported that CKD was associated with COVID-19-related death [40].

In our cohort, the median BMI was in the overweight range and a third of patients were obese. Despite this high percentage of patients in our cohort being overweight, nutritional risk screening is central because, during hospitalization, the nutritional status will likely deteriorate, as a study in Italy showed [41]. In that cohort of medical patients hospitalized with COVID-19, the mean unintentional weight loss was 7.6%. A large community-based study in the UK described obesity as a risk factor for severe COVID-19 [42]. Interestingly, the association between a higher BMI and mortality decreased in older-aged patients. Recinella et al. even suggested a higher BMI as a protective factor in elderly patients [43]. In this study with patients over 65 years, a higher BMI was associated with lower in-hospital mortality. They explained these findings with a higher BMI being an indicator for a better nutritional status in elder patients. 

In our study, lower albumin levels were associated with a higher risk for in-hospital mortality, ICU admission, and longer LOS. The association of low albumin levels with in-hospital mortality is in line with findings of two meta-analyses [44,45]. Even though, historically, albumin was thought to be a malnutrition marker, it is known that albumin is a negative acute phase protein [46]. Taking this into account, the association between hypoalbuminemia and mortality in COVID-19 patients is probably better explained by the inflammatory state and disease severity and not by the nutritional status. Furthermore, we found an association of low albumin levels and LOS, which was not only observed in patients with COVID-19. Bretscher et al. showed that, in a cohort of patients at nutritional risk, hypoalbuminemia was associated with longer LOS [21]. Lastly, hypoalbuminemia was associated with a higher risk for ICU admission, which was also found in a French study analyzing patients with COVID-19 [11].

This study has some limitations. First, our findings are limited to hospitalized patients in a single center. Our analysis is purely observational and we cannot make assumptions on whether the use of nutritional support would improve outcomes in COVID-19 patients as shown in medical patients [6,47] and patients with respiratory infections [48]. Further, due to the low patient number, only a low statistical power was achieved.

## 5. Conclusions

In conclusion, in patients with COVID-19, the risk for malnutrition was a risk factor for in-hospital mortality. Similarly, low albumin was associated with higher mortality and ICU admissions and with a longer hospital LOS. Future studies should investigate the role of nutritional treatment in this vulnerable patient population.

## Figures and Tables

**Table 1:** Baseline characteristics stratified by in-hospital mortality.

	Overall	Survivors	Non-Survivors	*p*-Value
	*n* = 305	*n* = 261	*n* = 44	
Sociodemographics				
Age, median (IQR)	66.0 (55.0, 75.0)	63.0 (53.0, 73.0)	74.5 (68.5, 80.0)	<0.01
Male gender, n (%)	203 (66.6)	170 (65.1)	33 (75.0)	0.20
Active smoker, n (%)	26 (12.4)	22 (12.6)	4 (11.4)	0.85
Comorbidities				
Age-adjusted Charlson comorbidity index, median (IQR)	3.0 (2.0, 5.0)	3.0 (1.0, 5.0)	5.0 (4.0, 7.0)	<0.01
Clinical Frailty Score, median (IQR)	3.0 (2.0, 4.0)	3.0 (2.0, 4.0)	4.0 (3.0, 5.0)	<0.01
Cancer, n (%)	36 (11.8)	22 (8.4)	14 (31.8)	<0.01
Hypertension, n (%)	167 (54.8)	141 (54.0)	26 (59.1)	0.53
Coronary artery disease, n (%)	63 (20.7)	47 (18.0)	16 (36.4)	<0.01
Chronic heart failure, n (%)	6 (2.0)	4 (1.5)	2 (4.5)	0.18
Asthma, n (%)	20 (6.6)	17 (6.5)	3 (6.8)	0.94
Chronic obstructive pulmonary disease, n (%)	23 (7.5)	16 (6.1)	7 (15.9)	0.02
Obstructive sleep apnea, n (%)	28 (9.2)	20 (7.7)	8 (18.2)	0.03
Solid organ transplant recipient, n (%)	7 (2.3)	7 (2.7)	0 (0.0)	0.27
Kidney transplant, n (%)	6 (2.0)	6 (2.3)	0 (0.0)	
Kidney–pancreas transplant, n (%)	1 (0.3)	1 (0.4)	0 (0.0)	
Active rheumatic disease, n (%)	7 (2.3)	5 (1.9)	2 (4.5)	0.28
Chronic kidney disease, n (%)	68 (22.3)	45 (17.2)	23 (52.3)	<0.01
Obesity (BMI > 30 kg/m^2^), n (%)	97 (32.1)	84 (32.6)	13 (29.5)	0.69
Diabetes, n (%)	81 (26.6)	68 (26.1)	13 (29.5)	0.63
Nutritional assessment				
BMI [kg/m^2^], median (IQR)	27.6 (24.2, 31.7)	27.6 (24.2, 31.7)	27.65 (25.2, 30,3)	0.96
Bodyweight [kg], median (IQR)	82.7 (70.8, 93.8)	82.9 (70.1, 94.2)	81.9 (72.9, 92.4)	0.95
NRS 2002				
NRS 2002 overall, median (IQR)	2.0 (1.0, 3.0)	2.0 (1.0, 2.0)	2.0 (2.0, 3.0)	<0.01
<3 points	229 (75.1)	205 (78.5)	24 (54.5)	<0.01
3–4 points	66 (21.6)	51 (19.5)	15 (34.1)	
≥5 points	10 (3.3)	5 (1.9)	5 (11.4)	
Initial vital signs				
Blood pressure, systolic [mmHg], median (IQR)	141.0 (128.0, 156.5)	141.0 (127.5, 156.0)	140.0 (130.0, 160.0)	0.79
Blood pressure, diastolic [mmHg], median (IQR)	81.0 (73.0, 89.0)	81.0 (73.0, 90.0)	78.0 (73.5, 87.5)	0.45
Pulse [bpm], median (IQR)	85.3 (77.0, 94.0)	85.0 (77.0, 94.0)	86.2 (77.9, 95.5)	0.66
Respiratory rate [breaths/min], median (IQR)	21.0 (17.5, 24.7)	21.0 (17.5, 24.4)	22.7 (17.9, 26.4)	0.21
Temperature [°C], median (IQR)	37.6 (36.8, 38.3)	37.6 (36.8, 38.2)	37.6 (36.8, 38.5)	0.67
SpO_2_ [%], median (IQR)	94.0 (90.1, 96.5)	94.3 (90.3, 96.6)	91.7 (84.3, 95.8)	0.02
Initial laboratory findings				
Haemoglobin [G/L], median (IQR)	134.0 (120.0, 145.0)	134.5 (120.0, 145.0)	130.0 (105.0, 140.5)	0.02
Leukocytes [G/L], median (IQR)	7.4 (5.1, 9.3)	7.3 (5.1, 9.2)	7.8 (5.1, 11.1)	0.41
Sodium [mmol/L], median (IQR)	137.0 (134.0, 139.0)	137.0 (134.0, 139.0)	138.0 (133.0, 139.5)	0.54
Glucose [mmol/L], median (IQR)	6.5 (5.7, 8.1)	6.4 (5.7, 7.9)	7.4 (5.8, 9.5)	0.12
Potassium [mmol/L], median (IQR)	3.8 (3.5, 4.1)	3.8 (3.5, 4.1)	4.0 (3.7, 4.2)	0.03
Calcium [mmol/L], median (IQR)	2.2 (2.1, 2.2)	2.2 (2.1, 2.2)	2.2 (2.1, 2.2)	0.66
Albumin [G/L], median (IQR)	30.2 (27.1, 33.7)	30.5 (27.6, 33.8)	28.0 (23.9, 31.7)	<0.01
Vitamin D [nmol/L], median (IQR)	50.1 (22.9, 57.1)	49.0 (26.8, 56.5)	57.1 (15.6, 75.9)	0.81
Creatinine [µmol/L], median (IQR)	91.0 (74.0, 113.0)	87.0 (72.0, 111.0)	111.0 (94.5, 170.5)	<0.01
Alanine-Aminotransferase [U/L], median (IQR)	35.0 (25.0, 50.0)	35.0 (25.5, 51.5)	33.0 (25.0, 50.0)	0.78
Alkaline phosphatase [IU/L], median (IQR)	69.0 (55.0, 92.0)	68.0 (55.0, 90.0)	77.0 (60.0, 115.0)	0.18
CRP [mg/L], median (IQR)	76.3 (28.6, 133.0)	71.0 (25.4, 122.0)	104.5 (64.6, 176.5)	<0.01
PCT [µg/L], median (IQR)	0.1 (0.1, 0.2)	0.1 (0.1, 0.2)	0.1 (0.1, 0.5)	0.01
In-hospital outcomes				
ICU care, n (%)	44 (14.4)	31 (11.9)	13 (29.5)	<0.01
Need for mechanic ventilation, n (%)	22 (7.2)	11 (4.2)	11 (25.0)	<0.01
Length of hospital stay [day], median (IQR)	7.0 (4.0, 13.0)	6.0 (4.0, 11.0)	14.5 (5.0, 20.5)	<0.01

Abbreviations: BMI, body mass index; CRP, c-reactive protein; ICU, intensive care unit; IQR, interquartile range; NRS, nutritional risk screening; PCT, procalcitonin; SpO_2_, oxygen saturation.

**Table 2:** Malnutrition parameters stratified by in-hospital mortality and crude and adjusted association of malnutrition parameters and in-hospital mortality.

	Survivors	Non-Survivors	*p*-Value	Crude OR (95% CI), *p*-Value	Adjusted OR * (95% CI), *p*-Value
	*n* = 261	*n* = 44			
NRS 2002					
NRS 2002 overall, median (IQR)	2.0 (1.0, 2.0)	2.0 (2.0, 3.0)	<0.001	1.63 (1.30, 2.10), *p* < 0.001	1.39 (1.07, 1.80), *p* = 0.013
NRS 2002 cut-offs, n (%)					
NRS 2002 < 3 points	205 (78.5)	24 (54.5)	<0.001	Reference	Reference
NRS 2002 3–4 points	51 (19.5)	15 (34.1)		2.51 (1.23, 5.13), *p* = 0.011	1.64 (0.76, 3.57), *p* = 0.204
NRS 2002 ≥ 5 points	5 (1.9)	5 (11.4)		8.54 (2.31, 31.65), *p* = 0.001	4.68 (1.18, 18.64), *p* = 0.029
BMI					
BMI overall, median (IQR)	27.6 (24.2, 31.7)	27.6 (25.2, 30.3)	0.96	1.00 (0.94, 1.06), *p* = 0.944	1.04 (0.97, 1.11), *p* = 0.247
BMI cut-offs, n (%)					
BMI < 20 if age < 70, BMI < 22 if age ≥ 70	16 (6.5)	3 (7.9)	0.75	Reference	Reference
BMI ≥ 20 if age < 70, BMI ≥ 22 if age ≥ 70	229 (93.5)	35 (92.1)		0.82 (0.23, 2.94), *p* = 0.755	1.64 (0.41, 6.53), *p* = 0.481
Albumin					
Albumin overall, median (IQR)	30.5 (27.6, 33.8)	28.0 (23.9, 31.7)	<0.001	0.90 (0.83, 0.96), *p* = 0.002	0.92 (0.85, 0.99), *p* = 0.030
Albumin cut-offs, n (%)					
<34.0 g/L	173 (75.5)	33 (82.5)	0.34	Reference	Reference
≥34.0 g/L	56 (24.5)	7 (17.5)		0.66 (0.27, 1.56), *p* = 0.341	0.98 (0.39, 2.45), *p* = 0.964

Abbreviations: BMI, body mass index; CI, confidence interval; IQR, interquartile range; NRS 2002, nutritional risk screening 2002; OR, odds ratio. * adjusted for gender and age-adjusted Charlson comorbidity index (ACCI).

**Table 3:** Malnutrition parameters stratified by ICU admission and crude and adjusted association of malnutrition parameters and ICU admission.

	No ICU Admission	ICU Admission	*p*-Value	Crude OR (95% CI), *p*-Value	Adjusted OR * (95% CI), *p*-Value
	*n* = 261	*n* = 44			
NRS 2002					
NRS 2002 overall, median (IQR)	2.0 (1.0, 3.0)	2.0 (1.0, 2.0)	0.66	0.98 (0.77, 1.24), *p* = 0.845	1.14 (0.87, 1.49), *p* = 0.335
NRS 2002 cut-offs, *n* (%)					
NRS 2002 < 3 points	193 (73.9)	36 (81.8)	0.091	Reference	Reference
NRS 2002 3–4 points	61 (23.4)	5 (11.4)		0.44 (0.17, 1.17), *p* = 0.100	0.61 (0.22, 1.70), *p* = 0.345
NRS 2002 ≥ 5 points	7 (2.7)	3 (6.8)		2.3 (0.57, 9.30), *p* = 0.244	3.58 (0.80, 16.11), *p* = 0.096
BMI					
BMI overall, median (IQR)	27.6 (24.0, 31.7)	27.9 (26.1, 31.4)	0.63	1.00 (0.95, 1.06), *p* = 0.993	0.99 (0.94, 1.06), *p* = 0.877
BMI cut-offs, *n* (%)					
BMI < 20 if age < 70, BMI < 22 if age ≥ 70	19 (7.8)	0 (0)	0.067	NA	NA
BMI ≥ 20 if age < 70, BMI ≥ 22 if age ≥ 70	224 (92.2)	40 (100)		NA	NA
Albumin					
Albumin overall, median (IQR)	30.4 (27.6, 34.0)	28.9 (26.0, 31.6)	<0.01	0.90 (0.84, 0.97), *p* = 0.003	0.88 (0.82, 0.95), *p* = 0.001
Albumin cut-offs, *n* (%)					
<34.0 g/L	171 (75.0)	35 (85.4)	0.15	Reference	Reference
≥34.0 g/L	57 (25.0)	6 (14.6)		0.51 (0.21, 1.29), *p* = 0.155	0.41 (0.16, 1.06), *p* = 0.066

Abbreviations: BMI, body mass index; CI, confidence interval; ICU, intensive care unit; IQR, interquartile range; NRS 2002, nutritional risk screening 2002; OR, odds ratio. * adjusted for gender and age-adjusted Charlson comorbidity index (ACCI).

**Table 4:** Crude and adjusted association of malnutrition parameters and length of hospital stay.

	LOS [Days], Mean (SD)	Unadjusted Coefficient (95% CI), *p*-Value	Adjusted Coefficient * (95% CI), *p*-Value
NRS 2002			
NRS 2002 overall		0.52 (−0.22, 1.26), *p* = 0.168	0.26 (−0.56, 1.07), *p* = 0.534
NRS 2002 cut-offs			
NRS 2002 < 3 points	8.83 ± 8.07	Reference	Reference
NRS 2002 3–4 points	7.47 ± 5.49	−1.36 (−3.72, 1.00), *p* = 0.258	−2.03 (−4.49, 0.43), *p* = 0.106
NRS 2022 ≥ 5 points	15.40 ± 9.32	6.57 (−0.26, 13.40), *p* = 0.059	4.77 (−2.14, 11.67), *p* = 0.175
BMI			
BMI overall		−0.10 (−0.26, 0.07), *p* = 0.244	−0.05 (−0.22, 0.12), *p* = 0.543
BMI cut-offs			
BMI < 20 if age < 70, BMI < 22 if age ≥ 70	8.44 ± 7.57	Reference	Reference
BMI ≥ 20 if age < 70, BMI ≥ 22 if age ≥ 70	8.79 ± 7.83	0.36 (−3.62, 4.34), *p* = 0.860	1.35 (−2.71, 5.40), *p* = 0.514
Albumin			
Albumin overall		−0.37 (−0.55, −0.20), *p* = 0.000	−0.34 (−0.52, −0.17), *p* = 0.000
Albumin cut-offs			
<34.0 g/L	8.62 ± 6.46	Reference	Reference
≥34.0 g/L	6.68 ± 5.91	−1.94 (−3.86, −0.02), *p* = 0.047	−1.58 (−3.54, 0.38), *p* = 0.114

Abbreviations: BMI, body mass index; CI, confidence interval; LOS, length of hospital stay; NRS 2002, nutritional risk screening 2002; SD, standard deviation. * adjusted for gender and age-adjusted Charlson comorbidity index (ACCI).

## Data Availability

The data presented in this study are available on request from the corresponding author.
